# Diagnostic difficulty in an adolescent with dissociative identity disorder

**DOI:** 10.4102/sajpsychiatry.v31i0.2333

**Published:** 2025-02-12

**Authors:** Kajal M. Patel, Luzuko Magula

**Affiliations:** 1 Department of Clinical Pharmacy, Faculty of Pharmacy, Rhodes University, Grahamstown, South Africa; 2 Department of Psychiatry and Behavioural Sciences, Faculty of Medicine and Health Sciences, Walter Sisulu University, Mthatha, South Africa; 3 Department of Psychology, Faculty of Humanities, Rhodes University, Makhanda, South Africa

**Keywords:** dissociative identity disorder, adolescent, alter, dissociate, multiple personalities

## Abstract

**Introduction:**

Dissociative identity disorder (DID) is a complex and controversial psychiatric condition characterised by the presence of two or more distinct identities, personality states, or identities that recurrently take control of an individual’s behaviour. The identities or personality states may have distinct characteristics, memories, and behaviours, making identifying and differentiating them challenging. We describe a complex case that presented diagnostic challenges because of the fluctuations in psychiatric presentations associated with DID, and we outline a multidisciplinary and biopsychosocial intervention.

**Patient presentation:**

A 15-year-old transgender female presented with psychosis, suicidal ideation, a history of self-harm and aggressive behaviour, and panic attacks. She had a diary with excerpts that she could not remember writing and a history of forgetting certain parts of her day. She displayed extreme variations of psychiatric presentations, including depression, mania, panic, and aggression.

**Management and outcome:**

The patient’s alters were individually treated based on their psychiatric presentation and theme. Management followed the phased approach of the International Society for the Study of Trauma and Dissociation (ISSTD guidelines), which included establishing safety and symptom reduction, integration of traumatic memories and identity as well as rehabilitation.

**Conclusion:**

In this case report, we present an adolescent with a myriad of psychiatric presentations and describe her management. We summarise key difficulties that a clinician can encounter in diagnosing DID.

**Contribution:**

We bring awareness to the complexity of this diagnosis. Lastly, we propose an Multidisciplinary team (MDT) biopsychosocial approach that helps to manage the condition.

## Introduction

Dissociative identity disorder (DID) is a complex and controversial psychiatric condition characterised by the presence of two or more distinct identities, personality states, or identities that recurrently take control of an individual’s behaviour.^1^ It is often covert and may go unnoticed or misdiagnosed for years.^2^ The identities or personality states may have distinct characteristics, memories, and behaviours, making identifying and differentiating them challenging.^2^ Many individuals with DID present with symptoms that are attributed to other conditions, such as depression, anxiety, personality disorders, substance use disorders, or posttraumatic stress disorder (PTSD).^3^ The disorder typically emerges as a response to severe childhood trauma, serving as a defence mechanism to protect the individual from overwhelming emotions or experiences.^4^

### Aims

We describe and bring awareness to a complex case that presented diagnostic challenges because of the fluctuations in psychiatric presentation associated with DID and outline a multidisciplinary and biopsychosocial intervention.

### Methodology

This is a single case design, explanatory study. Data were collected from clinical interviews with the patient and her parents, clinical notes and referral notes of the referring clinician, and the ward MDT ward interaction and their clinical notes. To ensure validity and reliability, all members of the MDT independently interviewed the patient and reviewed her symptomatology. Two clinicians collated the information independently during two ward rounds run by the different clinicians a week. The patient was closely observed and her behaviour was documented regularly. This correlated to the content written in the communication book the patient filled in. Psychiatric rating scales, including the Screen for Child Anxiety Related Disorders (SCARED), Children’s Yale-Brown Obsessive Compulsive Scale (CY-BOCS), Dissociative Experiences Scale (DES), Multidimensional Inventory of Dissociation (MID) and the Posttraumatic Stress Disorder Checklist for DSM-5 (PCL-5) were used to make a clinical assessment objectively and to quantify the severity of her symptoms.

## Ethical considerations

Ethical clearance to conduct this study was obtained from the Walter Sisulu University Health Sciences Research Ethics Committee (No. 027/2024). Approval to conduct the study was obtained from the hospital and the provincial department of health. The article was not read by the patient or the guardian, which is a limitation of this case study. Written and verbal informed assent and consent were obtained from the participants and their guardians.

## Patient presentation

### Case study

Patient X is a 15-year-old, English-speaking transgender female in Grade 8 who presented with suicidal ideation, agitation, self-harming behaviour, a history of aggressive behaviour and acute psychosis. A week before admission, the patient impulsively overdosed on 50 paracetamol tablets, triggered by thoughts of being bullied at school and being rejected by her father and brother because of her gender identity. The patient’s parents suspected that the patient killed the family cat in the same week and displayed agitation when asked about this. She complained of seeing a scary black creature with red eyes and sharp white teeth, which would instruct her to kill her mother. She reported seeing unfamiliar spirits hanging from the ceiling.

She reported spontaneous panic attacks triggered by reading passages in her diary with violent images of murder and death that she did not remember writing. In her diary, there were repeated diagrams of triangles, which she believed to be the symbol of a religion that she alone was a part of. She had drawn triangles on her thigh with a razor blade to remind herself of her ‘all powerful’ belief system. A SCARED questionnaire revealed severe anxiety, where she scored highest for panic disorder followed by generalised anxiety disorder and social anxiety disorder.

In the ward, staff noticed that the patient displayed idiosyncratic behaviours such as touching objects in multiples of three and separating her food items before consuming them. A CY-BOCS revealed a score of 20. Her obsessions stemmed from the fear of harm coming to herself and her mother, forbidden sexual thoughts, concern with her genitalia and violent and horrific images like those in her diary. The patient’s compulsions included multiple checks with her mother to make sure she was safe, opening and closing books three times and walking around tables in multiples of three.

The patient had episodes of 15 min to an hour of the day that she could not account for. A diagnosis of DID was made based on clinical history, observation and assessment based on the American Psychiatric Association’s (2022) Diagnostic and Statistical Manual of Mental Disorders (5th ed., text rev.; DSM-5-TR), the DES, and the MID. The alters documented by the patient between 11 July 2023 and 20 July 2023 are shown in Table 1.

**TABLE 1:** Alters documented by X.

Name of alter†	Description	Timing
X	‘Human. Host of the body. Generally calm and pleasant but struggles with emotions. Dissatisfied and reactive and paranoid’.	‘Generally, out most of the time’
K – G	‘Human. Five-year-old. Permanently scared of getting sexually assaulted again’. The patient reports this alter ‘shows her bad memories’.	Came out on 19 July 2023 from 17:40–17:55.
N	‘Human. Prone to panic attacks but a caretaker and is there often to intervene’. Patient reports that this alter ‘suffers most from Social Anxiety Disorder but is also down to earth’.	Came out on 18 July 2023 from 08:30–09:30.
S	‘Human. Depressed and empty and comes out whenever depressed’. Patient reports that this alter ‘makes depression worse and personifies emptiness’.	Usually comes out in the morning between 08:00 and 11:00, mostly midweek.
H	‘Human. Sounds calm but psychotic. Could be on a bad hallucinogen drug trip’. Patient reports this alter is ‘messy and disorganized with a paranoid mentality’.	Usually out during the day.
G	‘Human. Very calm, one with nature and interconnected with a pagan ritualistic thinking’. Patient reports that this alter keeps her safe, steady and coping.	Came out on 18 July 2023 between 09:30 and 10:30.
D	‘Confused, disorientated and obsessed with blood’. Not much is known about this alter.	Came out on 11 July 2023, between 19:20 and 19:40.
A	Patient describes this alter as ‘the Manic Alter’ because she is energetic and fun.	Comes out in the late morning or early evening.
P	‘A human. Fully devoted to taking care of me but blunt. Boasts superiority. Becomes foul-mouthed and does opposite of what others say. Becomes aggressive when engaged. Serious danger to everyone including self’.	This alter usually comes out after dark, when feeling ‘respected or glorified enough.’ Came out on 16 July 2023 between 18:00 and 19:00.

† , Names changed to alphabets for confidentiality.

Patient X’s past inpatient psychiatric history includes a three-week admission to an adolescent psychiatric unit in October 2022 for presenting symptoms of depression, anxiety, and psychosis and a 1-week admission in January 2023, where she presented with a psychotic episode and a history of decline in academic performance at school accompanied by social withdrawal. Past medications prescribed include Brexpiprazole, sodium valproate, fluoxetine, buspirone, olanzapine and quetiapine. There were minimal symptom responses and improvement with these, although compliance was uncertain. As an outpatient, she was diagnosed with autism spectrum disorder when she was 12 years old and was assessed as high functioning.

Patient X reported being allegedly sexually abused by her aunt when she was 5 years old. A clinical assessment was done and the PCL-5 assisted with a diagnosis of PTSD.

The patient’s mother is being pharmacologically treated for depression, and her father was diagnosed with attention deficit hyperactivity disorder as a child.

The patient was born at term as a result of an artificial insemination, with no obstetric complications reported. She had a speech impediment as a child and struggled with sensory stimulation like loud sounds, certain smells, and light touch. She did not have friendships, struggled to cope with being bullied at school and displayed obsessive interest in new hobbies every few months.

## Management and outcome

A physical examination, blood workup, lumbar puncture, and MRI brain scan were performed and were all normal. A distinct advantage in identifying the alters and the dissociation was the extensive documentation of the timing of her symptoms from the patient’s diary in collaboration with the nurse’s observations.

Following confirmation of the different alters, the MDT was able to observe different psychiatric presentations during the appearance of each. A biopsychosocial formulation was carried out, which identified psychological factors such as a longstanding history of parental conflict, dysfunctional peer relationships and poor coping skills, social factors such as unconfronted childhood trauma and disturbances in the family unit. Protective factors for this patient included an affinity for academics and a good maternal bond. Management of the patient was aligned with the International Society for the Study of Trauma and Dissociation (ISSTD) guidelines^5^ for DID. The initial treatment goal was to establish safety, stabilise the patient, and reduce the intensity of the symptoms.

The following measures were put in place to establish safety of the patient. A violent alter was de-escalated and/or sedated if needed. A depressed alter was treated using counselling and cognitive behavioural therapy (CBT) techniques. The patient and her family were psycho-educated on the diagnosis, and all the alters were introduced to them. Contingency plans and behavioural thresholds for hospitalisation were discussed for violent and suicidal alters.

To stabilise the patient, she was treated with Ziprasidone 40 mg orally at night, Buspirone 20 mg orally 8 hourly, and Fluoxetine 60 mg orally daily. The psychologist and occupational therapist equipped the patient with distress tolerance and emotional regulation skills and stressed the importance of accountability of the behaviours of the different alters. Further stabilisation techniques included extended leave of absences from the ward, where the patient was reintegrated into the family dynamic and symptomatic treatment was emphasised. On discharge, patient X had a decreased frequency of panic attacks, was able to emotionally regulate herself, which prevented alter P from appearing, had fewer psychotic episodes and the frequency at which alters appeared had decreased.

The second phase was implemented with regular psychotherapy, where the focus was confronting traumatic memories and learning to integrate them. Alter K – G and P were worked on predominantly as they were perceived to hold traumatic memories. The goal of this phase is to de-fragment the experienced trauma and assist the patient as an individual to tolerate difficult affects associated with the trauma such as anger, grief, shame and helplessness.

Reintegration and rehabilitation constitute the third phase of treatment where the patient is encouraged to develop a stable sense of self. This phase was not yet reached as it can take many years for the patient to integrate their alters. Rehabilitation was supported by establishing good communication between alters using a diary and having the patient join a home school where she was able to slowly continue her studies at her own pace.

## Discussion

Four important factors have contributed to the difficulty in diagnosing DID in the patient described above.

### Complex presentations and co-morbidities

One of the factors that makes it difficult to diagnose DID is its complex presentation and myriad of co-morbidities. Patient X presented with a list of varying symptoms such as depression, anxiety, psychosis, and dissociation. Studies correlate DID with patients who have been diagnosed with posttraumatic stress disorder, depression, and substance use disorders.^6^ There are opposing views regarding the trauma model that suggests that DID is a severe form of PTSD as it originates from severe childhood trauma, and the fantasy model suggests that DID is born from suggestibility and the patient’s fantasy world.^7^ This severe, fragmented dissociation can be seen in alter K – G, who feels as if she is being touched inappropriately, especially when falling asleep. There is also an association of DID with borderline personality disorder (BPD)^8^ and schizophrenia.^9^ Borderline personality disorder and DID share some core features, which include dissociation, a history of severe childhood trauma,^4^ and a disturbance in one’s sense of identity. A study conducted by Fung et al. (2023) showed that 43.3% of the participants with BPD displayed varying degrees of dissociation, the most severe being distinct identities that co-exist with their own emotions, memories, and behaviours.^10^ Patient X’s impulsive suicide attempt, her self-harm episodes and affective instability can also be explained by BPD. With regard to DID and schizophrenia, the first-rank symptoms that were thought to differentiate schizophrenia from other psychiatric disorders have now been shown to be shared by DID.^11^ Although patient X does not display first-rank symptoms with the exception of auditory hallucinations, she still fulfils the criteria for schizophrenia as per the DSM-5. From the aforesaid, it can be seen that patient X can be diagnosed with multiple psychiatric disorders, which was one of the main diagnostic challenges.

### Inadequate professional training

The incorrect and ignorant portrayal of DID in the media, together with the uncertainty and a lack of adequate knowledge as well as scrutiny of clinicians, has led to the underdiagnosis of DID in the community.^12^ Studies show that patients receive an average of four preceding diagnoses and that it takes approximately 6.8 years to receive the correct diagnosis of DID. This can lead to years of health-related costs, personal suffering, and multiple trials of failed medications.^12^ Patient X had two prior admissions where she was incorrectly treated for psychosis, anxiety and depression and had been on multiple treatment regimens with little symptom resolution. During the most recent admission, staff initially had difficulty containing the fluctuations in presentation, as this was the first time some staff members were exposed to DID. Staff were educated on the topic and were trained on recognising triggers and understanding different alters.

### Neurochemistry and non-specific symptoms

There are brain imaging studies that may be able to identify ‘biomarkers’ of DID.^13^ Studies show that cortical volume, thickness, surface area,^14^ and global hippocampal volume^15^ were decreased in DID patients compared to healthy controls. This abnormal brain morphology was correlated to early childhood trauma. This makes it non-specific as this may be similar in patients with PTSD. These ‘biomarkers’ were not present in patient X’s MRI. Not all patients with PTSD or DID will present with identifiable changes on brain imagining, and if they do, the lack of specificity presents a challenge to diagnostic clarity.

### Malingering

Dissociative symptoms can be malingered at times to assume the sick role, to attain financial benefit, or to circumvent the legal system, particularly in forensic or state patients.^16^ There is also a possibility of the fantasy model of trauma coming into effect where genuine symptoms are exaggerated. There was a concern among the MDT that the patient X is suggestible and may find comfort in assuming the sick role. Rates of malingering can be as high as 17% in forensic settings and 7% in psychiatric institutions.^16^ The Infrequency Psychopathology Scale of the Minnesota Multiphasic Personality Inventory (MMPI-2)^17^ and the Structured Interview of Reported Symptoms (SIRS-2)^18^ have good specificity to differentiate DID from simulators.

### Recommendation

We encourage screening for DID and careful exploration of the dissociative episodes in complex presentations of a fluctuating nature with multiple diagnoses and behavioural disturbances coupled with dissociative and amnestic phenomena.
